# Monitoring Sintering Burn-Through Point Using Infrared Thermography

**DOI:** 10.3390/s130810287

**Published:** 2013-08-09

**Authors:** Rubén Usamentiaga, Julio Molleda, Daniel F. Garcia, Francisco G. Bulnes

**Affiliations:** Department of Computer Science and Engineering, University of Oviedo, Campus de Viesques, Gijón, Asturias 33204, Spain; E-Mails: jmolleda@uniovi.es (J.M.); dfgarcia@uniovi.es (D.F.G.); bulnes@uniovi.es (F.G.B.)

**Keywords:** sintering burn-through point, infrared thermography, active contour segmentation, feature extraction

## Abstract

Sintering is a complex industrial process that applies heat to fine particles of iron ore and other materials to produce sinter, a solidified porous material used in blast furnaces. The sintering process needs to be carefully adjusted, so that the combustion zone reaches the bottom of the material just before the discharge end. This is known as the burn-through point. Many different parameters need to be finely tuned, including the speed and the quantities of the materials mixed. However, in order to achieve good results, sintering control requires precise feedback to adjust these parameters. This work presents a sensor to monitor the sintering burn-through point based on infrared thermography. The proposed procedure is based on the acquisition of infrared images at the end of the sintering process. At this position, infrared images contain the cross-section temperatures of the mixture. The objective of this work is to process this information to extract relevant features about the sintering process. The proposed procedure is based on four steps: key frame detection, region of interest detection, segmentation and feature extraction. The results indicate that the proposed procedure is very robust and reliable, providing features that can be used effectively to control the sintering process.

## 1. Introduction

Sintering is a primary process in steel production that applies heat to fine particles of iron ore to transform them into a coarse-grained product [1]. This is a very complex process in which many chemical reactions occur at the same time. Therefore, monitoring sintering is a challenging task. However, temperature can be a very good indicator about the behavior of the sintering process.

Temperature measurement and monitoring is a mandatory requirement for most steps in steel manufacturing [2]. Measuring, monitoring and controlling steel temperature throughout the manufacturing process ensures that materials meet product specifications, preventing defects in the final product [3,4]. Temperature can also be used to detect objects, where images taken in the visible spectrum are not adequate [5].

Sintering applies a heat treatment process to the material. Thus, temperature measurement and monitoring during and after the sintering process are critical to achieve optimal results [6]. Only if the heat treatment is correct will sintering produce the desired results. Inappropriate control of the sintering process may lead to cracking, distortion and other defects in the final product [7].

During the sintering process, a mixture of fine particles of iron ore and other materials, such as coke and limestone, are charged onto a set of sintering pallets and leveled to form a bed. Then, the material is ignited. Combustion begins in the top layers of the mixture and moves down through the bed, while the sintering pallets move forward. The sintering process needs to be carefully adjusted, so that the flame front reaches the bottom of the bed, known as the burn-through point (BTP), just before the discharge end. The BTP must be near a desirable set-point at approximately the discharge end, so that adequate fusion of sintering materials occurs.

Many different parameters need to be finely tuned in the sintering process. Control is mostly based on models that calculate the inputs of the process depending on predictions. Different techniques are used, including fuzzy logic, neural networks, finite state machines and genetic algorithms [8–13]. All these models require precise feedback to fine-tune the parameters.

This paper presents a sensor-to-monitor sintering BTP based on infrared thermography. It uses an infrared camera to acquire 2D infrared images from the material at the discharge end of the sintering process. The sequence of images is processed in order to detect the most appropriate images to monitor this process. Only some of these images provide relevant information about the process, those where the position of the flame front can be clearly seen. Thus, the first step is to identify these images. Then, the selected images must be analyzed to extract different features to provide feedback for the process control. The system needs no configuration; it automatically detects the material in the images and the regions of interest. The information provided by the system can be used to control many parameters of the sintering process, including the strand speed, the bed height, the quantity of coke, water and other materials in the mixture.

The proposed system has been installed in one of the two sintering machines of ArcelorMittal in Gijón, Spain. Sinter production in this plant is about 3,000,000 tons of product per year.

This paper is organized as follows: Section 2 describes the sintering process; Section 3 presents the proposed approach for sintering BTP monitoring; Section 4 discusses the results obtained and finally, Section 5 reports conclusions.

## 2. The Sintering Process

Sintering is an agglomeration process in which iron ore fines and other products such as coke fines are mixed and fired at a temperature high enough to achieve a certain degree of vitrification. This process produces a solidified porous material known as sinter. The initial mixture is made up of particles with a diameter smaller than 3 mm, and the resulting sinter has a diameter of approximately 30–60 mm. Blast furnaces that produce pig iron use sinter as one of the principle raw materials.

**Figure 1.**
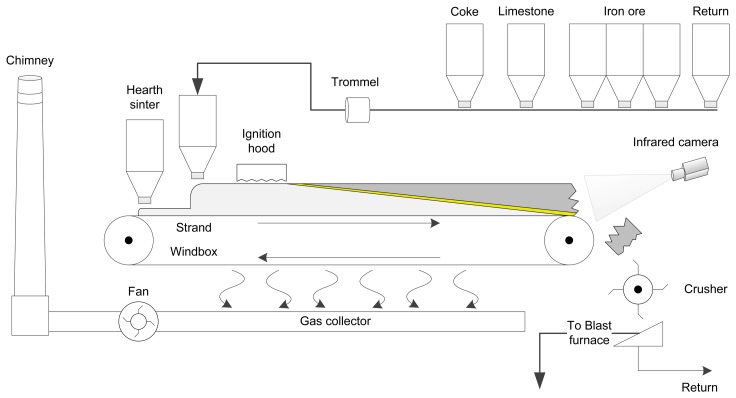
Sintering process using a Dwight-Lloyd machine.

The most common machine used for sintering is the Dwight-Lloyd machine. Figure 1 shows a schematic representation of the sintering process using this type of machine. The sinter is prepared in a continuous process. Different materials are meticulously mixed: iron ore fines are mixed with other materials such as limestone and coke, which is used as fuel. Water is also added to the mixture to increase the relative humidity. Furthermore, included in the mixture are pieces of sinter, which have been returned for reprocessing, because they were too small. The mixture is loaded onto a layer of hearth sinter (coarse sinter) on a moving strand and leveled to form a bed. The mixture is moved using sintering pallets on rails. The sintering mixture forms a sintering bed that passes under an ignition hood, where the fine coke in the upper layer is ignited by gas flames.

The hot gas produced by the combustion is sucked in through the material from the wind boxes placed below. Combustion begins in the top layers and moves down through the bed, due to the burning air that flows throw the ignited bed from the top towards the bottom of the bed. A portion of the material in the high temperature region, known as the flame front, melts. This partial melting causes the particles to agglomerate [14]. It takes between 15 and 20 min for the flame front of the mixture to reach the bottom layer of the bed, depending largely on permeability. Strand speed must be controlled, so that sintering is completed before the discharge end. The BTP, the point where the flame front reaches the bottom of the bed, must occur near a desirable set-point very close to the discharge end. Only this way will fusion of sintering materials be correct.

The resulting solidified sinter is crushed and screened to a size suitable for a blast furnace feed. Material of acceptable size is transported in a conveyor belt to the blast furnace to proceed to the next step of the steel-making process. Undersized material is recycled back to the process.

**Figure 2.**
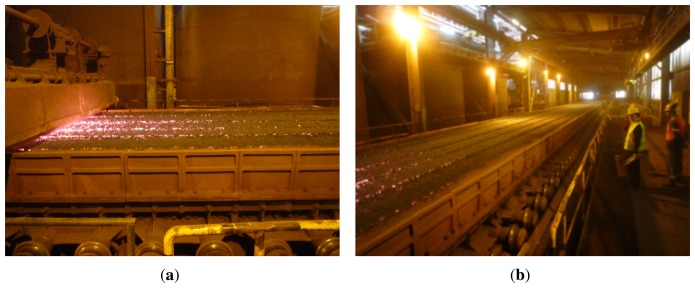
Ignition hood and sinter bed. (**a**) Mixture bed just after the ignition hood; (**b**) mixture bed on the sintering pallets.

Figure 2 shows two images of the sintering process. Figure 2(a) shows the mixture just after the ignition hood. Figure 2(b) shows the sinter bed. As can be seen, the mixture is moved in a big structure, around one hundred meters long and three meters wide.

## 3. Proposed Approach

**Figure 3.**
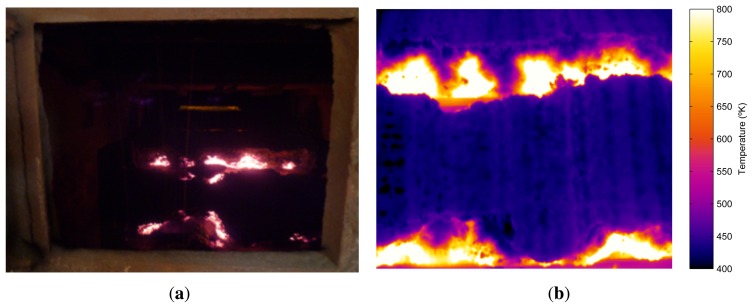
Discharge end of the sintering process. (**a**) Image taken in the visible spectrum; (**b**) infrared image.

The camera used in the system is at the discharge end of the sintering process. The position of the camera can be seen in Figure 1. Figure 3(a) shows an image taken from this position in the visible spectrum. This image shows the flame front in the material, although in images taken in the visible spectrum, the position is barely appreciable. In contrast, Figure 3(b) shows an infrared image taken from the same position, where the flame front can be seen much more clearly. These images show why it is necessary to use infrared thermography. A sensor based on infrared thermography is not only able to measure the temperature of the flame front, but can also be used to detect the position of the flame front. This position is extremely important to determine the BTP and to regulate the process parameters that can be used to optimize sintering. The infrared camera used in this system is a Flir ThermoVision A325, which provides infrared images of 320 × 240 pixels. The maximum acquisition frequency of the camera is 60 Hz, *i.e.*, a new image can be acquired every 16.67 ms. The temperature range is configurable within several available ranges. In the experiments, the range, [300, 2,000 °K], was selected. The manufacturer reports a measurement accuracy of ±2°K and a sensitivity lower than 50mK. The camera communicates with the computer using a dedicated Gigabit Ethernet cable. The long-wave infrared camera operates at eight to 12 μm. The camera is configured with an emissivity of 0.9, an estimated value for rough iron and porous materials, and adjusted using other readings.

Temperature measurement with infrared devices is largely affected by the emissivity of the material. Emissivity calibration is especially complicated in low emissivity objects, such as polished steel or aluminum, because small variations in emissivity lead to large variations in the resulting temperatures. However, in objects with high emissivity, such as a sintering mixture, slight variations in the chosen emissivity value cause only minor changes in the resulting surface temperatures.

The movement of the material is slow compared to the maximum acquisition rate of the camera. For this application, it is not necessary to use a high frame rate. An acquisition rate of 15 frames per second provides more than enough resolution to obtain images with the best possible view of the flame front.

**Figure 4.**
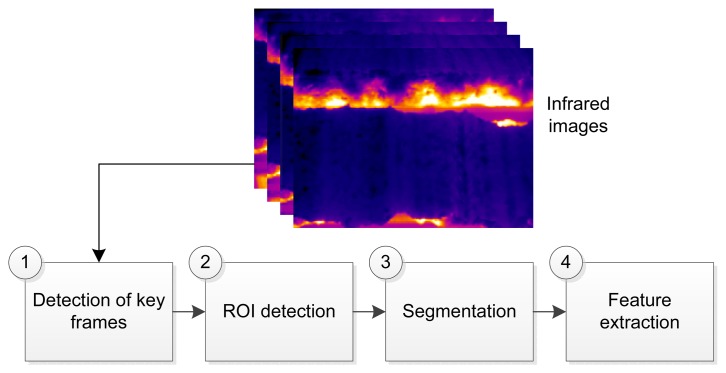
Summary of the proposed approach for sintering burn-through point (BTP) monitoring.

The proposed approach for sintering BTP monitoring is broken down into several steps, which are outlined in the following sections. Figure 4 shows a summary of the steps.

### 3.1. Detection of the Key Frames

**Figure 5.**
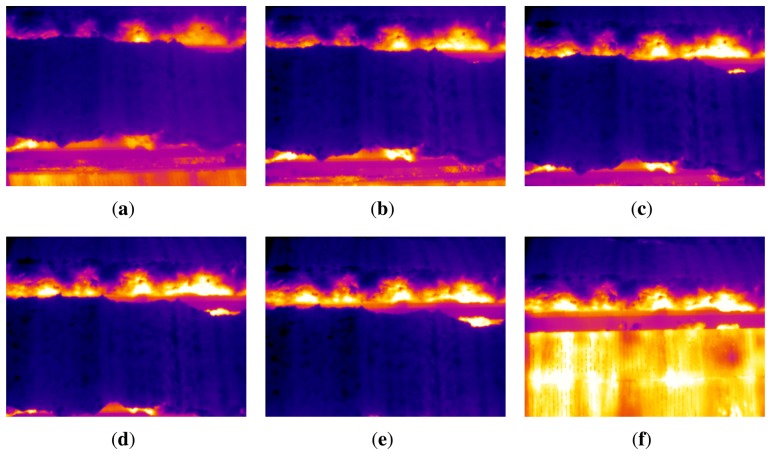
Infrared images acquired while the sinter is being discharged. (**a**) Image at *t* = 0; (**b**) image at *t* = 1.6 s; (**c**) image at *t* = 3.2 s; (**d**) image at *t* = 4.8 s; (**e**) image at t = 6.4 s; (**f**) image at *t* = 8 s key frame where the flame front is clearly visible.

The system acquires images from the discharge section of the sintering process. Not all the images acquired contain useful information. Most of them show the top layer of the sinter, and the flame front is hidden. Thus, it is necessary to identify the images where the flame front is visible from the sequence of infrared images. These images are called key images, since they are the key to extracting relevant features about the BTP in the sintering process. Figure 5 shows several consecutive images acquired while the sinter is being discharged. As can be seen, the material moves forward until it falls into the crusher located a few meters below. At the beginning, the flame front is hidden under the sinter. However, as the point where the sinter falls into the crusher gets closer, the flame front starts to become visible in the images. The flame front is clearly seen just after the sinter falls. At this point, the part of the material that is falling stops hiding the flame front. Therefore, this would be the best image to extract features about the flame front and to determine where the BTP occurred.

When the sinter is discharged, the temperature of the top layer of the sinter is much lower than the temperature of the sintering pallets used to move the material. Thus, when the sinter falls and the sintering pallets become visible, the average temperature in the infrared image is much higher. This can be seen in Figure 5. These images are shown using the same color scale as in Figure 3(b). Temperature in the sintering pallet is similar to the temperature in the flame front, while temperate in the top layer of the sinter is several hundred degrees lower. Figure 5(f) shows a key frame, where the sinter has just fallen, and the flame front is clearly visible. Furthermore, the average temperature in this frame is higher than the others. As the sinter moves, the sintering pallet will gradually be hidden again, and the average temperature of the image will decrease. Thus, the average temperature of the images is a good indicator to identify key frames.

**Figure 6.**
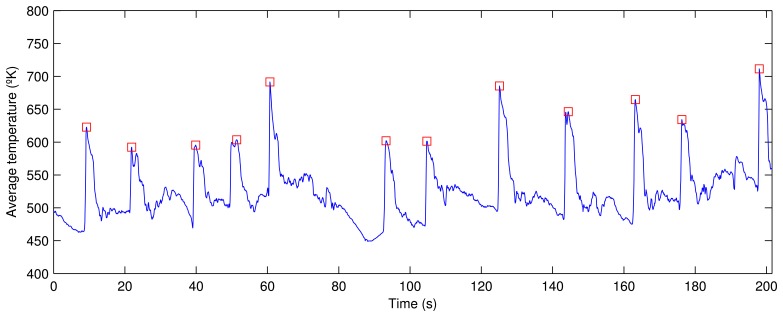
Average temperature per frame at 15 frames per second.

Figure 6 shows the average temperature per frame over a period of three and a half minutes. In the resulting signal, several peaks are clearly appreciable. These peaks correspond to frames where the sinter has just fallen, that is, these are the key frames. In other words, the key frames can be detected by detecting the peaks in the average temperature signal.

In order to detect the peaks in the average temperature signal, a procedure based on derivatives is used. The first step is to calculate the first derivative of the signal. Possible peaks are detected, because the first derivative of a peak has a downward-going zero-crossing at the peak maximum. However, the presence of random noise causes many false zero-crossings. Possible peaks are filtered based on two conditions: the slope must exceed a minimum, and the original signal at that point must exceed another minimum value. In this way, small peaks are ignored. Figure 6 shows all the peaks detected in the average temperature signal using this procedure. The proposed procedure is very robust and provides very good results under different conditions. When this procedure is applied in real time, a very small delay is necessary to calculate the first derivative with a window centered around each point, but this does not affect the results.

**Figure 7.**
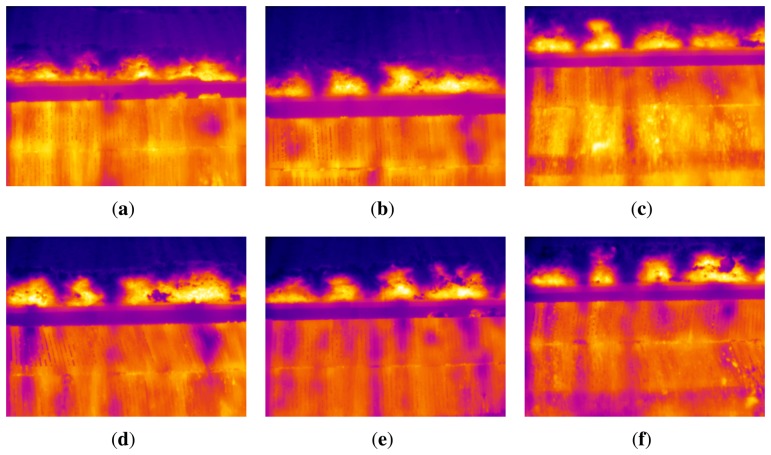
Examples of detected key frames. (**a**) Frame 1; (**b**) Frame 2; (**c**) Frame 3; (**d**) Frame 4; (**e**) Frame 5; (**f**) Frame 6.

The position of the peaks in the average temperature signal makes it possible to identify the key frames in the sequence of infrared images. The position of a peak corresponds to a key frame. Figure 7 shows some of these key frames. These are the images where the flame front is best seen and, thus, the images used for the subsequent processing. Figure 7(f) will be used as an example to describe further processing.

### 3.2. Detection of the Region of Interest

The position of the flame front is not always at the same level. This is because the sinter does not always fall from the same position, as it varies with the density and porosity of the mixture. Thus, it is necessary to identify the region in the image where the flame front is located.

Region of interest (ROI) is the commonly used term to refer to the portion of an image of particular interest. In this case, the ROI is the portion of the image where the flame front is located. The part of the image where the sintering pallet is located is not of interest and must be removed. Once the ROI is defined, there is no need to process the whole image to detect the position of the flame front. Thus, the computational cost is greatly reduced.

The ROI in the image is located above the sintering pallet. In the moving strand, there are many sintering pallets moving on rails. When the pallet reaches the discharge end of the sintering process, it rotates, and the sinter falls into the crusher. It is at this point where the key frames are detected. When the pallet rotates, a space appears between consecutive pallets that can be seen in Figure 7. This space appears in all the key frames and can be used as a limit between the ROI (the part of the image above) and the part of the image with no interest for processing.

**Figure 8.**
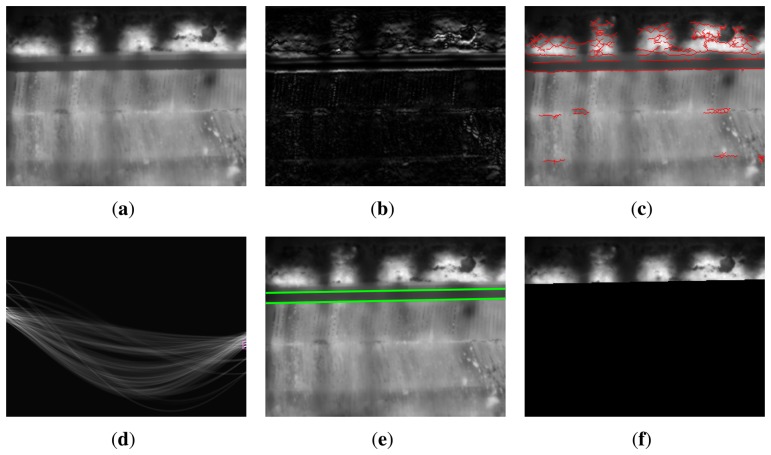
Detection of the regions of interest. (**a**) Key frame; (**b**) absolute value of the gradient; (**c**) edges after thresholding, thinning and opening; (d) Hough transform; (**e**) lines detected in the image; (**f**) final region of interest after removing the unwanted area.

ROI detection starts by applying a gradient detector to a key frame. The gradient of the infrared image is calculated vertically using the method proposed in [15], which is significantly more accurate than previous methods. The gradient is only calculated vertically, because the space between sintering pallets appears as a semi-horizontal fringe in the image. Figure 8(b) shows the resulting gradient using this method for the image shown in Figure 8(a).

Next, the absolute value of the gradient is thresholded. Two different morphological operations are applied to the resulting binary image: thinning and opening. Thinning is an operation used for skeletonization and reduces all lines to single-pixel thickness. Opening is an operation used to remove small objects. Pixels are connected, and the objects that have fewer than a specific number of pixels are removed. The connectivity matrix used to decide if two pixels are connected is shown here:

$$(\begin{array}{ccc} 1 & 0 & 1\\ 1 & 1 & 1\\ 1 & 0 & 1 \end{array})$$

This connectivity matrix is selected, because it connects horizontal lines as valid objects, but not vertical lines. The final result of this process can be seen in Figure 8(c). This figure shows the resulting edge map over the original image. It can be seen that the edges of the space between pallets are clearly detected.

The Hough transform is applied next to the edge map. The Hough transform is the most commonly used method for line detection in an image, because it can be used without prior knowledge and under extreme noise conditions [16]. In the Hough transform, pixels are mapped to lines in a discretized 2D parameter space using radius-angle parameterization. By restricting the angle parametrization to specific ranges of valid angles, only some of the lines of the image are detected [17]. Figure 8(d) shows the Hough transform. The two points highlighted in this image correspond to the two lines shown in Figure 8(e). The angle parametrization has been restricted in order to search for only these two lines: the top and bottom limits of the space between sintering pallets.

The top line seems to be a good division between the part of the image where there is interesting information and the part of the image that must be removed. However, careful examination reveals a small gap between the top line and the bottom of the sinter bed. The real line should be a few pixels above the detected top line. However, the distance from the top line to the bottom of the sinter bed depends on the position of the top line. Due to the position of the camera, when the position of the top line is low in the image, the distance is greater than when the position is high in the image. This issue can be solved by creating a small lookup table that indicates the required distance depending on the position of the top line. This table is created off-line and depends on the relative position between the camera and the scene.

Using the top line detected by the Hough transform and the described lookup table, a new line is calculated: this line corresponds to the bottom of the sinter bed in the image. Figure 8(e) shows the ROI in the image, that is, the part of the image above this line. The rest of the image is removed and will be ignored in the next steps.

### 3.3. Segmentation of the Frame Front

Image segmentation divides the image into sets of segments with similar attributes, in this case, temperature. Segmentation is one of the oldest and most widely studied aspects of computer vision [18]. However, non-uniformities and blurry edges in the images make many traditional image analysis routines unsuitable for infrared image processing [19,20]. This is why many applications process infrared images using *ad hoc* algorithms [21–23].

For segmentation of the flame front in infrared images, many methods (such as thresholding, edge detection, region growing or watershed) fail or provide inaccurate results. The proposed method for this task is an image segmentation based on active contours models [24,25], using a region-based active contour model.

There are many advantages of active contour models over classic image segmentation methods. For example, active contour models can achieve sub-pixel accuracy of object boundaries and can incorporate prior knowledge, such as shape and intensity distribution. They also provide smooth closed contours as segmentation, which can be used for shape analysis and recognition. Particularly important is their ability to perform in images with intensity heterogeneities, which often occur in real images taken from both the visible and the infrared spectrum.

Active contour segmentation aims to minimize an energy function by evolving the current contour towards image features. The energy function is defined based on the specific active contour model [26]. The original model is based on three forces: internal, external and image forces. Internal forces give the model tension and stiffness. External forces come from human operators or initialization procedures. Images forces are driven by image features, such as light and dark regions. The total energy of the model can be represented as Equation (1), where *x* (*s*) represents the position of the contour parametrically.

(1) $$E=\int_{0}^{1}E_{\text{element}}(x(s))ds$$

The total energy can be rewritten in terms of the three basic energy functions as Equation (2).

(2) $$E=\int_{0}^{1}E_{\text{int}}(x)ds+\int_{0}^{1}E_{\text{ext}}(x)ds+\int_{0}^{1}E_{\text{img}}(x)ds$$

Existing active contour models can be divided into two major classes: edge-based models and region-based models. Edge-based models use local edge information to evolve the contour toward the object boundary using, for example, an energy function based on a two-dimensional spline curve. Region-based models use an energy function based on a certain region descriptor, which guides the motion of the active contour.

In this work, the precise segmentation of the flame front is carried out using a region-based active contour model in a variational level set formulation [27]. This active contour model defines a region-scalable fitting energy function in terms of a contour and two fitting functions that locally approximate the image intensities on the two sides of the contour. This segmentation method can be used to segment images with intensity heterogeneity and also with weak object boundaries. The flame front in the infrared images considered in this work has weak boundaries. Furthermore, the temperature in this flame front could vary in different parts of the image, provoking intensity heterogeneities in the image. Thus, the segmentation method fits this problem perfectly.

**Figure 9.**
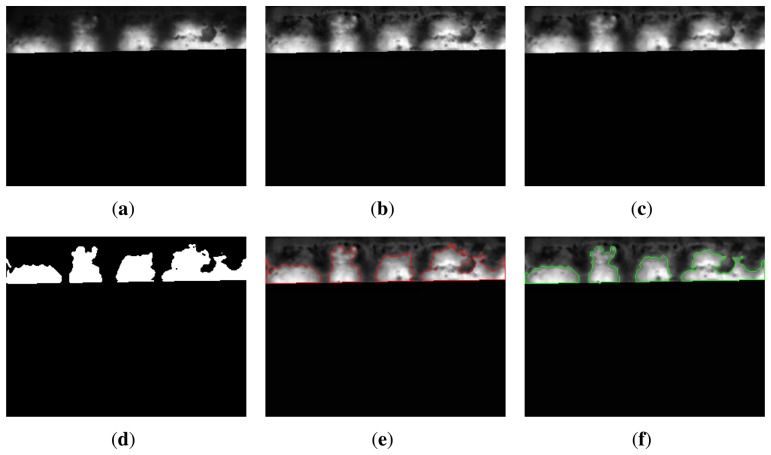
Segmentation of the flame front. (**a**) Input image; (**b**) image after contrast enhancement; (**c**) median filtering of the image; (**d**) binary image after thresholding; (**e**) initial contours; (**f**) final segmentation.

Before carrying out segmentation based on active contour, it is necessary to prepare the image. First, a contrast enhancement procedure is applied to the image [28]. This procedure improves the contrast in the image, making the edges of the flame front sharper. Figure 9(b) shows the result of this procedure for the image shown in Figure 9(a). The resulting image is then filtered using the median with a small window size (3 × 3). This procedure removes noise and improves the results of the segmentation using active contours. The resulting image is now ready for the segmentation of the flame front. The image after median filtering can be seen in Figure 9(c).

Segmentation using active contours requires the specification of an initial contour. In this case, the initial contours are established by applying a thresholding process [29]. The resulting binary image after image thresholding is a rough approximation for the segmentation of the flame front. However, it is a good starting point to continue the processing and obtain a final adjusted boundary.

Figure 9(d) shows the binary image obtained after thresholding. The boundaries of this mask represent the initial contours. Figure 9(e) shows the starting point of the active contour segmentation. The initial contours evolve towards an accurate segmentation of the flame front. The final result in Figure 9(f) clearly identifies the regions of the flame front in the image. In this case, the initial and final contour do not differ greatly. This is because the temperature of the flame front is homogeneous, and the thresholding is able to detect it very well. Nevertheless, the boundary of the region on the right is much more adjusted and accurate in the final image.

### 3.4. Feature Extraction

In general, the objective of feature extraction is to characterize an object by measurements whose values are very similar for objects in one category and very different from objects in others [30]. This leads to the idea of seeking distinguishing features. However, the goal in this work is to measure one or more of the properties of the material, so that its quality can be assessed; in this case, the quality based on the BTP obtained from the information about the flame front at the end of the sintering process. These features can then be used as feedback to fine-tune the parameters of the sintering process. Thus, different features that provide information about the BTP are explored and analyzed.

#### 3.4.1. Sinter Discharge

The first set of features is related to how the sinter is discharged. In order to extract these features, the information about the detection of key frames and ROI is sufficient: segmentation of the flame front is not required.

The frequency at which the sinter mixture falls is an important feature. The value of this feature depends on the speed of the strand. However, it also depends on other parameters of the sintering process. This feature can be calculated as the number of peaks in the average temperature per frame in a period of one minute. In the example shown in Figure 6, the value of this feature is 3.63. A high value in this feature indicates an early disintegration of the sinter mixture. On the other hand, a low value indicates that the sinter mixture is too solid. In either of these two cases, the sinter mixture is not correct, and the amount of coke and water needs to be adjusted to change the moisture content and agglomeration of the material.

A complementary feature related to the same issues is the position of the line that divides the region of interest. When this line is at the top of the image, the sinter mixture disintegrates easily. If this line is towards the bottom of the image, the sinter mixture is stuck to the sintering pallet. These issues are also related to problems in the mixture.

#### 3.4.2. Hot Zone

The hot zone is the maximum temperature zone in the flame front. The position of this zone compared with the bottom of the sinter bed is a good indication of the BTP. In this case, interesting features are extracted by subtracting the position of the maximum temperature in the flame front and the position of the line that divides the region of interest.

**Figure 10.**
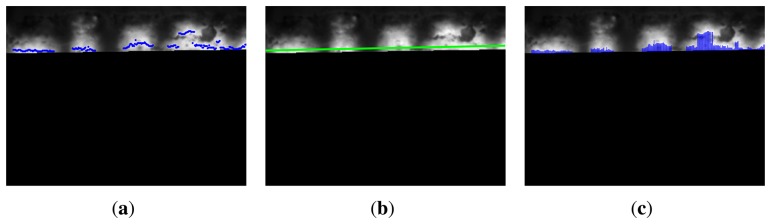
Maximum temperature zone in the flame front. (**a**) Position of the maximum temperature values in the flame front; (**b**) values fitted to a line; (**c**) distance from the values to the bottom limit of the region of interest (ROI).

Figure 10(a) shows the position of the maximum temperature values in the flame front. Ideally, they should all be part of a straight line parallel to the the bottom limit of the ROI. In this case, the set of points could be fitted to a line (Figure 10(b)), and the coordinates of this line would be the features. However, as can be seen in the images, not all the points lie on this line. A better approach is to measure the distance from each of these points to the the bottom limit of the ROI, as shown in Figure 10(c).

**Figure 11.**
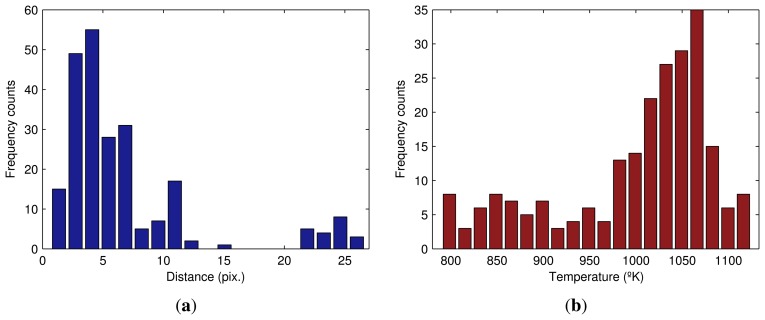
Histograms for the hot zone. (a) Histogram of the distance from the maximum temperature values in the flame front to the bottom limit of the ROI; (**b**) histogram of the maximum temperature values in the flame front.

The best way to analyze the distribution of a set of points is with a histogram. Figure 11(a) shows the histogram of the distance from the maximum temperature values in the flame front to the bottom limit of the ROI. The frequent distances are very close to zero. However, some values on the right-hand section of the figure indicate spurious behavior. The BTP should have occurred before the discharge end of the sintering process, but in this part of the flame front, BTP has yet to occur. This means that in this zone, some of the material has not received proper heat treatment.

The maximum temperature in the flame front is also a very good quality metric of the sintering process. Figure 11(b) shows the histogram of the maximum temperature values in the flame front.

Many useful features can be extracted from the histograms with the distribution of distances and temperature, such as, for example, the center of the data or the spread. It can also be used to calculate the skewness and the presence of outliers and multiple modes. All these features are of utmost importance to improve the result of the sintering process.

#### 3.4.3. Flame Front Zone

**Figure 12.**
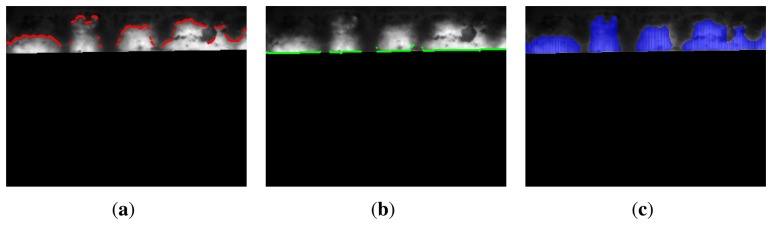
Heights in the flame front. (**a**) Top position. (**b**) bottom position. (**c**) heights.

**Figure 13.**
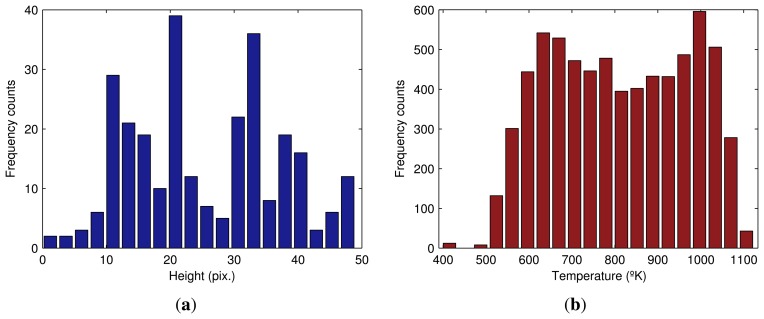
Histograms for the flame front zone. (**a**) Histogram of the heights in the flame front; (**b**) histogram of the temperature values in the flame front.

Another set of features can be extracted from the flame front zone. The first set of features is related to the heights of the zone. The heights of this zone can be computed from the binary image produced by the segmentation. The top and bottom limits of the zone are calculated (Figure 12(a,b)). Then, the distance is computed finding the difference between them (Figure 12(c) and Figure 13(a) show the histogram of the heights in the flame front).

The temperature distribution in the flame front is also important. Figure 13(b) shows the histogram of the temperature values in the flame front. This histogram can be compared with Figure 11(b), which only uses maximum temperature values. As can be seen, the shape of the histogram in this case is completely different. Features from both histograms can provide a full view of the temperatures at the end of the sintering process.

These two new histograms for the flame front zone provide a new set of features that complement features extracted from the hot zone. A similar procedure could be used to calculate numerical values from the obtained distributions.

The width distribution of the flame front is another central feature. Uneven distribution of the flame front across the width of the sinter bed indicates anomalies in the sintering process. It is especially useful to measure the width of the area where the flame front is missing. In the key frame used in this example, the value of this feature is 12.5%.

## 4. Results and Discussion

The proposed image processing and feature extraction procedure has been applied to an extensive dataset, containing thousands of infrared images. These experiments were used to adjust the parameters of the system and to validate the procedure. The proposed method provided robust detection of the key frames and the region of interest. However, in some cases, the line that divides the region of interest was very low in the image. In these cases, there is not an adequate perspective of the flame front in the image. These images are discarded, as they cannot be used to extract useful information. Most key frames show a perfect view of the flame front. In these images, the proposed segmentation procedure makes it possible to identify the position of the flame front in the image very accurately. The proposed procedure was very robust and effective.

**Figure 14.**
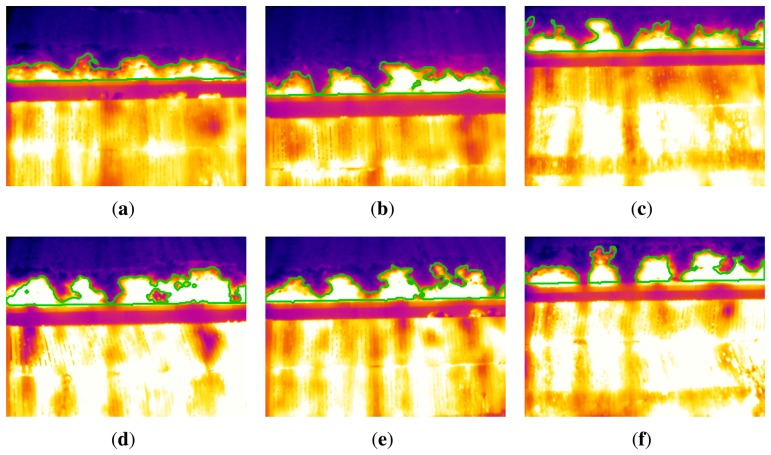
Results for the segmentation of the flame front in all key frames. (a) Frame 1; (**b**) Frame 2; (**c**) Frame 3; (**d**) Frame 4; (**e**) Frame 5; (**f**) Frame 6.

Figure 14 shows the segmentation results for the key frames shown in Figure 5. The ROI has been detected correctly. It is the bottom line of the flame front. From this division of the images, the segmentation procedure detects the boundary of the flame front accurately. The final results clearly identify the flame front in the infrared image.

**Figure 15.**
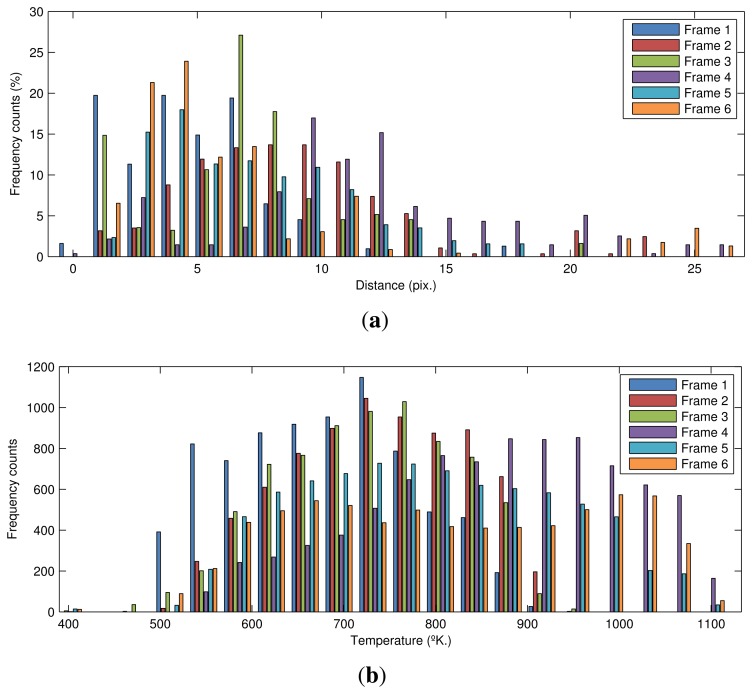
Histograms for the flame front zone in all key frames. (a) Histogram of the distance from the maximum temperature values in the flame front to the bottom limit of the ROI; (**b**) histogram of the temperature values in the flame front.

The final step of the proposed procedure is the extraction of features. Figure 15(a) shows a comparison of the distribution of distances from the maximum temperature values in the flame front to the bottom limit of the ROI for each key frame. In most of the frames, the distance is very low. As expected, at the end of the sintering process, the flame front is very close to the bottom of the sinter bed. However, there are cases, such as Frame 6, where there are long distances that could indicate anomalies in some areas of the sinter bed. Comparing these distributions can help to assess the BTP and to tune the sintering parameters.

Figure 15(b) shows a comparison of the distribution of temperature in the flame front for each key frame. The distribution is quite similar in all frames, with an average value around [700–800 °K] and a maximum around [1,000–1,100 °K]. Outliers in temperature distribution can indicate an incorrect mixture of the materials. In particular, high temperature can indicate an excess of coke.

The information obtained after processing the infrared thermography can be used to extract relevant features about the sintering process with high accuracy and reliability. The extracted features can be used to monitor not only the BTP, but also the sinter qualities. Furthermore, real-time information about the sintering process can be used to fine-tune the operation parameters, including the strand speed, the solid fuel rate and the return fine rate.

## 5. Conclusions

This paper proposes a method to monitor the sintering burn-through point based on infrared thermography. The proposed procedure acquires infrared images from the discharge end of the sintering process. These infrared images contain the cross-section temperatures of the sintering mixture. The first step is to detect the frames where the flame front can be seen best. This detection is based on the average temperature of the frames. The next step detects the region of interest in the images. In this case, a space between the sintering pallets is used as a reference in the images. A segmentation procedure is applied to the detected region of interest. A state-of-the-art active contours technique is used to identify the boundary of the flame front in the images. Finally, different features that can provide useful feedback for sintering control are analyzed and explored. No user input is required for any of the steps: a great advantage for deployment and maintenance.

Results indicate that the proposed system is very robust and reliable. Not only can the extracted features be used to monitor the sintering burn-through point, they can also be effectively used to monitor many other aspects of the sintering process or to predict sinter qualities. Furthermore, the provided information can be used to adjust the quantity of coke in the mixture, that is, the solid fuel rate. Therefore, the integration of this sensor in a sintering plant makes for a far more cost-efficient industrial process.
